# Impact of atorvastatin reload on the prevention of contrast-induced nephropathy in patients on chronic statin therapy: A prospective randomized trial

**DOI:** 10.1371/journal.pone.0270000

**Published:** 2023-05-08

**Authors:** Rania Hammami, Omar Masmoudi, Jihen Jdidi, Mouna Turki, Rim Charfi, Imtinene Ben Mrad, Amine Bahloul, Tarek Ellouze, Rania Gargouri, Samir Kammoun, Selma Charfeddine, Fatma Ayedi, Leila Abid

**Affiliations:** 1 Cardiology Department, Hedi Chaker Hospital, University of Medicine, University of Sfax, Sfax, Tunisia; 2 Epidemiology Department, Hedi Chaker Hospital, University of Medicine, Sfax, Tunisia; 3 Biochemistry Department, Habib Bourguiba Hospital, University of Medicine, Sfax, Tunisia; 4 Cardiology Department Habib Thameur Hospital, Tunis, Tunisia; Federico II University, ITALY

## Abstract

**Background:**

This trial aimed to assess the efficacy of Atorvastatin reloading on the prevention of Contrast-induced nephropathy (CIN) in patients pre-treated with this statin and undergoing coronary catheterization.

**Methods:**

This was a prospective randomized controlled study including patients on chronic atorvastatin therapy. We randomly assigned the population to the Atorvastatin Reloading group (AR group), by reloading patients with 80 mg of atorvastatin one day before and three days after the coronary procedure, and the Non-Reloading group (NR group), including patients who received their usual dose without a reloading dose. The primary endpoints were the incidence of cystatin (Cys)-based CIN and Creatinine (Scr)-based CIN. The secondary endpoints consisted of the changes in renal biomarkers (Δ biomarkers) defined as the difference between the follow-up level and the baseline level.

**Results:**

Our population was assigned to the AR group (n = 56 patients) and NR group (n = 54 patients). The baseline characteristics of the 2 groups were similar. Serum creatinine (SCr)-based CIN occurred in 11.1% in the NR group, and in 8.9% in the AR group without any significant difference. Cys-based CIN occurred in 37% in the NR group and 26.8% in the AR group without any significant difference. The subgroup analysis showed that high dose reloading had significantly reduced the CYC-based CIN risk in patients with type 2 diabetes (43.5% vs 18.8%, RR = 0.43. CI 95% [0.18–0.99])). The comparison of “Δ Cystatin” and Δ eGFR between the AR and NR groups didn’t show any significant difference. However, cystatin C had significantly increased between baseline and at 24 hours in the NR group (0.96 vs 1.05, p = 0.001), but not in the AR group (0.94 vs 1.03, p = 0.206).

**Conclusions:**

Our study did not find a benefit of systematic atorvastatin reloading in patients on chronic atorvastatin therapy in preventing CIN. However, it suggested that this strategy could reduce the risk of CyC-based CIN in diabetic type 2 patients.

## Introduction

Contrast-induced nephropathy (CIN) is a common complication occurring in 5 to 11% of angiographic procedures [1–4]. The CIN incidence has varied widely across studies as its definition is not the same throughout the literature [5]. CIN is the third leading cause of hospital-acquired acute kidney injury (AKI), accounting for 12% of all cases, next to hypovolemia (42%) and postoperative renal injury (18%) [6]; it accounts for up to 30% of acute kidney injury in hospitalized patients [7]. This complication is strongly associated with significantly increased mortality, extended hospitalization periods, and additional costs [8]. Given the increase in the number of coronary intervention procedures in the last decades and the difficulties to determine CIN mechanisms, many protective measures have been assessed in randomized and observational studies. The most known predictors of CIN occurrence according to the literature are advanced age, diabetes, use of angiotensin-converting enzyme inhibitor/angiotensin receptor blocker before the procedure, high baseline creatinine, the contrast type, hypertension, and left ventricular systolic dysfunction [1, 3, 7, 9]. Paradoxically, Statin pretreatment has been identified as a protector against CIN after PCI in observational studies [10, 11]. Recently, Randomized Controlled Trials (RCTs) including statin naïve patients showed that a loading dose of statin before catheterization reduces the risk of CIN significantly [12, 13]. In a recent large meta-analysis of 124 trials and 28240 patients, comparing 10 strategies of CIN prevention: saline, statin, and other strategies like N-acetylcysteine (NAC), sodium bicarbonate (NaHCO_3_), NAC+NaHCO_3_, ascorbic acid, xanthine, dopaminergic agent, peripheral ischemic preconditioning, and natriuretic peptide; compared with saline, the risk of CIN was significantly reduced by using statin (odds ratio [OR] = 0.42; 95% confidence interval [CI], 0.26–0.67). The benefit of statin therapy was consistent across multiple sensitivity analyses, whereas the efficacy of all the other strategies was questioned by restricting the analysis to high-quality trials [14]. Thus, the ESC guidelines on myocardial revascularization have already supported pre-treatment with high dose statins in statin-naïve patients since 2018, to prevent CIN (Class IIa, evidence level A) [15]. Up to date, there is still no evidence about the impact of statin reloading in patients on chronic statin therapy. Moreover, in most trials, we used a glomerular filtration rate (GFR) assessed by creatinine blood level. However, unlike cystatin C, creatinine is significantly affected by muscle mass (hence, sex or age), race, or diet. Furthermore, many studies proved that cystatin C, a small protein produced throughout the body by all nucleus cells, could be a more reliable and sensitive marker of kidney function and could be potentially used to generate a more precise estimate of GFR than creatinine [16–18]. Thus, we aimed in this trial to assess the impact of an additional loading dose of atorvastatin in patients who had already been on this drug on CIN incidence using measurement of Cystatin C level in blood.

## Methods

### Study population

This was an interventional prospective, randomized, single-blind, controlled trial, implemented in all consecutive patients (older than 18 years), undergoing coronary angiography or percutaneous coronary intervention in our department between June 2020 and September 2020 and who had already been receiving atorvastatin for at least one week, before admission.

We didn’t include in the present study patients with the following criteria: patients admitted because of an acute coronary syndrome in whom a loading dose is mandatory according to guidelines, statin-naïve patients, patients who received a statin other than atorvastatin before the procedure, patients already receiving 80 mg atorvastatin, patients requiring dialysis and those with eGFR less than 15 ml/min/ 1.73 m2, patients who were exposed to a Contrast Medium (CM) within 7 days, patients with an allergy to contrast media, patients with cardiogenic shock or severe cardiac insufficiency (left ventricular ejection fraction LVEF <20%), patients with severe liver damage, malignant tumor, infectious disease, or fever, and those who refused to consent. We also excluded the patients who didn’t return to get control laboratory tests.

The regional ethics committee (The Committee of Protection of the Persons in the South of the country: CPP South) approved the study and all participants signed the written informed consent.

The protocol of the study was registered on the Pan-African Clinical Registry (PACTR). The registration number of the study is: PACTR202110707328144.

### Study protocol

We randomly assigned all patients to either the Atorvastatin Reloading group (AR group) or the Non-Reloading group (NR group) according to a computer-generated random series of numbers. The randomization occurs one day before the coronary procedure. Patients in the AR group received oral atorvastatin 80 mg daily one day before and then 3 days after contrast media administration, followed by their habitual dose; patients assigned to the NR group received their habitual dose (atorvastatin 40 mg, 20 mg, 10 mg) without an additional reloading dose.

In accordance with the ESC guidelines, we suspended the nephrotoxic drugs one day before the procedure (aldosterone antagonists, inflammatory inhibitors) in all patients. The renin-angiotensin inhibitors and metformin were withheld if patients showed a moderate CKD (defined by an eGFR <60 mL/min/1.73 m²) [15].

All patients were treated with intravenous hydration with isotonic saline (0.9% sodium chloride) for 12 hours before and 12 hours after the procedure at the rate of 1 ml/Kg/H (0.5 ml/kg/H if LVEF <40% or if the patient suffered from dyspnea) and received the same nonionic dimeric iso-osmolar Contrast Media (CM) (Iopromide. ULTRAVIST 300 (300 mg d’Iode/mL). The nurses performed drug delivery and hydration. This designed study was single-blind. The physician who performed the coronary procedure was blinded to the patient’s group, but the patient was aware of his group.

The nurses (3 females) collected demographics, clinical, and biological data for all patients: age, gender, body mass index (BMI), cardiovascular risk factors, co-morbidities, clinical presentation, the kind of procedure (coronary angiography or PCI), left ventricle systolic function, and current medication. No relationship was established prior to the study commencement. The interview was not repetad. The details of the procedure were also documented. All the data were collected in a CRF that was not returned to the participant. The participants didn’t provide feedback on the findings. Data saturation was not discussed.

### Laboratory parameters

Blood samples were collected to measure the baseline values of Serum Creatinin (SCr), Cystatin C (Cys), inflammatory factors (high sensitive C-reactive protein [hsCRP]), and pro-BNP on admission, and of course, before the loading dose administration. The post-procedural levels of Cys were measured 24 hours after the coronary procedure, the SCr, and the hsCRP at 72 hours. Previous studies had shown that the peak of cystatin C elevation occurred by the 24^th^ hour, unlike SCr the peak occurred between the 48^th^ and 72^nd^ H [19]. Serum levels of creatinine were measured enzymatically and GFR was estimated by the MDRD formula (male: eGFR- Scr = 186×(serum creatinine)−1.154×(age)−0.203; female: GFR = 186×(serum creatinine)−1.154×(age)−0.203×0.742). Serum cystatin C was measured by nephelometry and eGFR-Cys was estimated by GFR = [74.835/(serum cystatin C (mg/l))] ^1.333^ [20].

### Study end-points and definitions

The primary endpoints were the incidence of Cys-based CIN defined as an increase in serum CyC concentration by 10% above the baseline value 24 hours after contrast media administration [18] or the incidence of SCr-based CIN defined as the increase in SCr concentration of 44.2 mmol/L or 25% above baseline within 72 hours after exposure to contrast media according to the KDIGO definition [21].

The secondary end-point was to detect any acute kidney injury by a significant rise in cystatin C level between baseline and 24 hours in the two groups (Δ cystatin).

The changes in renal biomarkers (Δ biomarkers) are defined as the difference between the follow-up level and the baseline level. These changes were compared between the two groups (AR and NR groups).

We assessed the risk of CIN before the procedure using the Mehran score [22]. It was first described by Roxana Mehran et al, in 2004, in a randomized study including 8351 patients undergoing PCI and aiming to determine predictors of CIN after PCI [22]. This score includes eight parameters (hypotension, intra-aortic balloon pump, congestive heart failure, chronic kidney disease, diabetes, age >75 years, anemia, and volume of contrast) and predicts the incidence of CIN with good discriminative power (*c*statistic = 0.67) [22]. The risk of CIN was considered low if Mehran’s score = 0 to 5, moderate if Mehran’s score = 6 to 10, and high if Mehran’s score>10.

### Statistical analysis

Statistical analyses were carried out using SPSS software version 23 (SPSS Inc., Chicago. Illinois, the USA). We expressed categorical variables as percentages, and continuous variables as mean values (±standard deviation [SD]) when the distribution was normal or medians with semi-interquartile ranges (SIQR) when it was not normal. Normally distributed continuous variables were compared using the Student t-test (independent sample t-test for comparison between the 2 groups, paired sample t-test for self-comparison); non-normally distributed continuous variables were analyzed by non-parametric test (Mann-Whitney U test for independent series and Wilcoxon test for paired series). When the application conditions were validated, categorical data were analyzed using the Chi2 test of Pearson, otherwise Fisher exact test.

Given the lack of similar studies in the literature, we carried out a pre-survey on 20 patients to determine the number of subjects needed. It was speculated that the incidence of CyC-based CIN was 36% in the NR group. We hypothesized the additional loading dose of Atorvastatin in the AR group could reduce the incidence of CyC-based CIN to 15%. Thus, the calculated sample size was at least 51 individuals for each group to get 80% power with a significance level of 0.05 (for the unilateral test). We have included an additional 10% of the workforce calculated taking into account the risk of loss of follow-up.

The number needed to treat to prevent one event was calculated according to this formula: NNT = 1/ARR with ARR = Absolute Risk Reduction.

## Results

**Fig 1:**
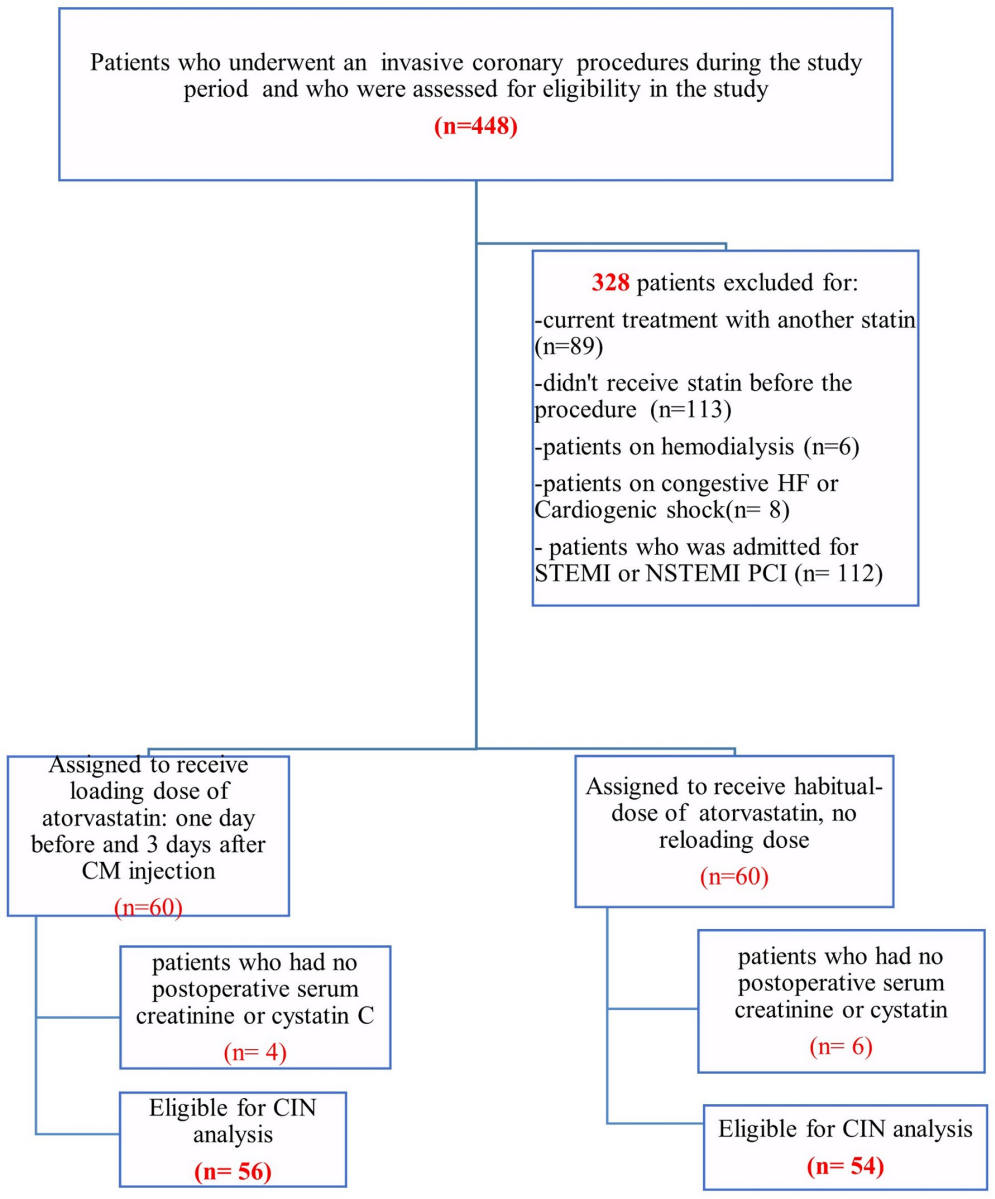
Trial flowchart.

Four hundred forty-eight patients underwent coronary procedures during the study period. According to inclusion criteria, 120 patients were randomly assigned to the Atorvastatin Reload group (AR group, n = 60) and the Non-reloading group (NR group, n = 60) (Fig 1). However, only 110 patients returned to undergo control laboratory tests; hence, we included only 56 patients in the AR group and 54 patients in the NR group. The number of included patients per day was low, less than one patient per day, given the COVID-19 pandemic, the refusal of certain patients to return for a control test, and the exclusion of acute coronary syndromes from our population. The number of planned coronary catheterization procedures was significantly reduced during this period.

Our population was at high cardiovascular risk, 58.2% of patients were smokers, 60.9% had hypertension, 51.8% had diabetes, 81% had dyslipidemia, 7.3% had moderate CKD, and 34.5% had a history of coronary disease. Sixty-six percent of our patients had at least three cardiovascular risk factors. All patients had been receiving atorvastatin for more than one month before the coronary catheterization (58.2% received 40 mg of atorvastatin, 7.3% received 20 mg of atorvastatin, and 34.5% received 10 mg of atorvastatin).

The baseline clinical characteristics and laboratory results are summarized in Tables 1 and 2.

**Table 1:** Baseline clinical characteristics in our population.

	Atorvastatin reload group (N = 56)	No Reload group (N = 54)
Age, years [mean± SD]	60.8 ±8.7	62.2 ±8.7
Age ≥ 70 years [n (%)]	8 (14.3)	12 (22.2)
Male [n (%)]	38 (67.9)	41 (75.9)
Body mass index (Kg/m^2^) [median. (SIQR)]	27.6 (3)	25.8 (2.5)
Obesity [n (%)]	19(39)	11(20.4)
Hypertension [n (%)]	35 (62.5)	32 (59.3)
Diabetes [n (%)]	34 (60.7)	23 (42.6)
Type 2 Diabetes [n (%)]	32 (57.1)	23(42.6)
Smoking [n (%)]	16 (28.6)	16 (29.6)
Dyslipidemia [n (%)]	42 (75)	48 (88.9)
Cv Risk factors number ≥3 [n (%)]	41(73.2)	32 (59.2)
Dysthyroidia [n (%)]	4(7.1)	3(5.6)
History of coronary disease [n (%)]	19 (33.9)	19(35.2)
Chronic Renal Failure [n (%)]	17 (30.4)	22(40.7)
NYHA Class		
NYHA I [n (%)]	28(50)	22(40.7)
NYHA II [n (%)]	25(44.6)	29 (53.7)
NYHA III [n (%)]	3(5.4)	3(5.6)
History of Contrast injection [n (%)]	14 (25)	16 (29.6)
Proteinuria on UD [n (%)]	6(10.7)	6(11.1)
Systolic blood pressure (mmHg) [mean± SD]	132.2 ±12.8	131.7 ±17.1
Diastolic blood pressure (mmHg) [mean± SD]	74.7 ±10.4	73.0 ±10.3
LVEF (%)	56.29±5	56.5±7.8
Previous dose of atorvastatin		
40 mg [n (%)]	36(64.3)	28(51.9)
20 mg [n (%)]	4(7.1)	4(7.4)
10mg [n (%)]	16(28.6)	22(40.7)
Performed procedure		
Coronary angiography [n (%)]	41(73.2)	38(70.4)
PCI [n (%)]	15(26.8)	16(29.6)
CM volume (mL) [Median.(SIQR)]	60 (20)	60 (15)
Adhoc PCI [n (%)]	14 (25)	8(14.8)
Contrast volume (Adhoc PCI)	110 (17.5)	105 (12.5)
[Median.(SIQR)] (n = 22)		
Mehran Score distribution		
Low-risk score [n (%)]	39 (69.6)	43 (79.6)
Moderate-risk score [n (%)]	14 (25)	9 (16.7)
High-risk score [n (%)]	3 (5.4)	2 (3.7)
Medications		
ACE inhibitors/ARBs [n (%)]	32 (57.1)	29 (53.7)
Diuretics [n (%)]	5 (8.9)	6 (11.1)
Anti-aldosterone [n (%)]	1(1.8)	3(5.6)
Digoxin [n (%)]	0(0)	1(1.9)
Beta-blockers [n(%)]	33(58.9)	38(70.4)
Antithrombotic therapy		
Aspirin [n (%)]	50(89.3)	39(72.2)
Clopidogrel [n (%)]	6(10.7)	9(16.7)
Acenoucoumarol [n (%)]	1(1.8)	5(9.3)
Antidiabetic drugs		
Metformine [n (%)]	32(57.1)	20(37)
Insulin [n (%)]	7(12.5)	7(13)

ACE: angiotensin-converting enzyme, CM: Contrast Media, Cv: cardiovascular, LVEF: Left Ventricle Ejection Fraction, NYHA: New York heart association, PCI: Percutaneous Coronary Intervention, SD: Standard deviation, SIQR: semi-interquartile range, UD: urine dipstick

**Table 2:** Baseline clinical laboratory tests in our population.

	Atorvastatin reload group (N = 56)	No Reload group (N = 54)
Cystatin C (mg/dl) [median. (SIQR)]	0.941 (0.18)	0.966 (0.16)
ProBNP (pg/ml) [median. (SIQR)]	63.15(51)	68.4 (92)
hsCRP (mg/dl) [median. (SIQR)]	1.6 (2.35)	1.25 (1.35)
Hemoglobin (g/dl) [median. (SIQR)]	13.5 (0.85)	13.5 (0.7)
Total cholesterol (mmol/l) [median. (SIQR)]	3.55 (0.75)	3.25 (0.85)
LDL cholesterol (mmol/l) [median. (SIQR)]	1.8 (0.66)	1.88 (0.61)
Creatinine (μmol/l) [Median.(SIQR)]	66 (11.5)	71 (16)
eGFR ml/min/1.73 m^2^ [Median.(SIQR)]	98 (17.5)	100(27)
eGFR		
≥90 ml/min/1.73 m^2^ [n (%)]	40(71.4)	33(61.1)
60-89ml/min/1.73 m^2^ [n (%)]	15(26.8)	14(25.9)
30–59 ml/min/1.73 m^2^ [n (%)]	1(1.8)	6(11.1)
15–29 ml/min/1.73 m^2^ [n (%)]	0(0)	1(1.9)
ASAT (UI/l) [median. (SIQR)]	15.9(5.2)	15.7 (5.45)
ALAT (UI/l) [median. (SIQR)]	14.2(6.85)	11.5(7.3)
CK [median. (SIQR)]	78.5(43)	76(39.5)
LDH (U/l) [median. (SIQR)]	171.5(42)	180(50.5)

Data are expressed as median (SIQR: semi-interquartile range).

ALAT: alanine aminotransferase ASAT: aspartate aminotransferase, CK: creatine kinase levels, LDH: Lactate dehydrogenase, LDL: Low-density cholesterol, eGFR: estimated glomerular filtration rate;, hsCRP: high-sensitivity C-reactive protein; intervention, ProBNP: Pro Brain natriuretic peptide

**Fig 2:**
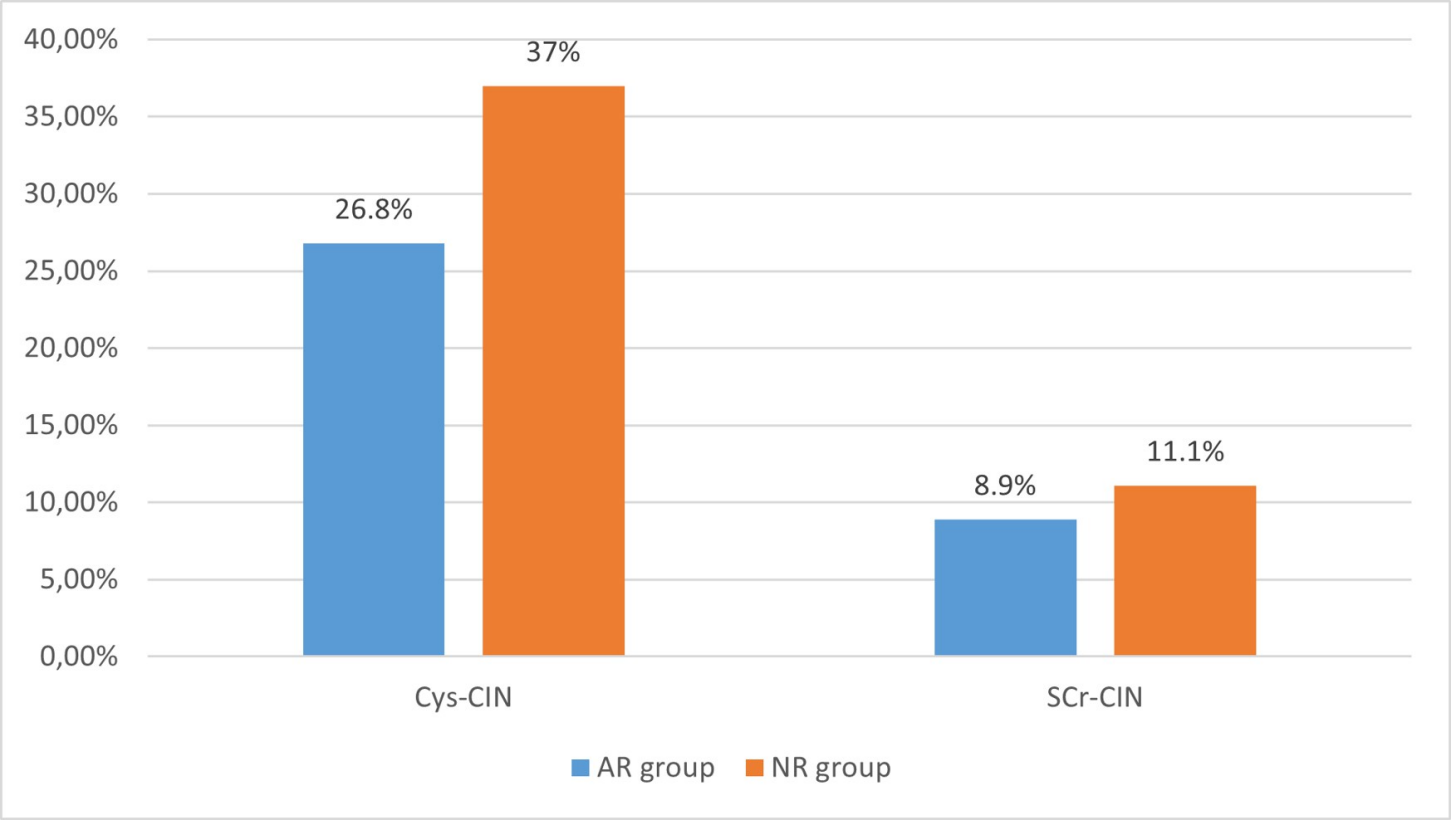
Comparison of contrast induced nephropathy between groups. AR group: patients who received a reloading dose of 80 mg atorvastatin, Cys-CIN: Contrast-induced nephropathy based on cystatin C levels, NR group: patients who did not receive a reloading dose, Scr-CIN: contrast induced nephropathy based on Serum creatinine levels.

The primary end-point of SCr-based CIN occurred in 11 patients (10%). There was no statistical difference in Scr-based CIN incidence between the AR group and the NR group (11.1% versus 8.9%, p = 0.7). The overall incidence of CyC-based CIN was higher than Scr-based CIN and it was calculated to be 31.8% (35/110) of the patients. This incidence was 37% in the NR group and 26.8% in the AR group without any statistical difference between the two groups (p = 0.24) (Fig 2).

**Fig 3:**
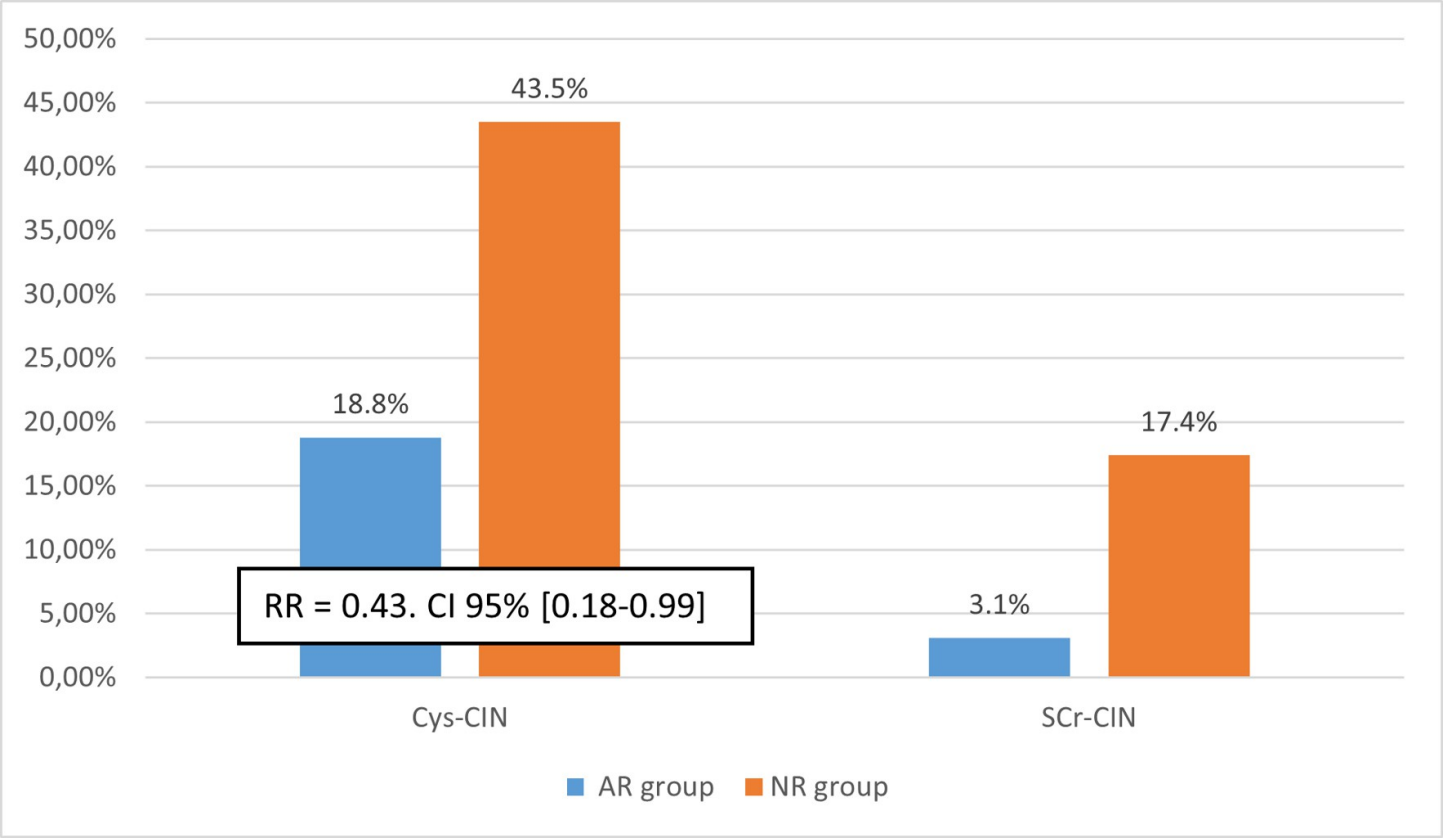
Comparison of contrast induced nephropathy between the two groups in diabetes 2 patients (n = 55). AR group: patients who received a reloading dose of 80 mg atorvastatin, Cys-CIN: Contrast-induced nephropathy based on cystatin C levels, NR group: patients who did not receive a reloading dose, Scr-CIN: contrast induced nephropathy based on Serum creatinine levels.

The analysis of subgroups who are known as a high-risk CIN population (patients with renal failure, patients aged > 70 years, type 2 diabetes, patients with Mehran score > 10, patients with heart failure, patients on ACE/ARBs) showed a benefit of additional atorvastatin reloading only in the type 2 diabetes group. The CyC-based CIN in patients with type 2 diabetes was significantly lower in patients who received a reload dose of atorvastatin than those didn’t receive a reload dose of statin (18.8% vs 43.5%,p = 0.046, RR = 0.43, Confidence Interval 95% [0.18–0.99]) (Fig 3). Thus, we had to treat 4 patients with high-dose reload of atorvastatin one day before the CM injection and one day after the procedure to prevent 1 case of CIN (number needed to treat: NNT = 1/ARR = 1/(0.435–0.188) = 4.

Paradoxically, SCr-based CIN was similar between the AR group and the NR group even in type 2 diabetic people.

When comparing changes of renal biomarkers (Δ cystatin, Δcreatinine and Δ Cys-eGFR) between the AR group and the NR group, there were no significant differences (Table 3). The comparison of renal biomarkers’ changes according to the previous dose of atorvastatin didn’t show any significant differences, either (Table 3).

**Table 3:** Comparison of changes in renal biomarkers between the AR and the NR groups.

	All The population			Previous Atorvastatin dose before randomization (10–20 mg)			Previous Atorvastatin dose before randomization (40mg)		
	AR group N = 56	NR group N = 54	p	AR group N = 20	NR group N = 26	p	AR group N = 36	NR group N = 28	p
ΔCreatinin (μmol/L) [median. (SIQR)]	4 (5.5)	1.5 (5.5)	0.14	1.5 (3.2)	0.5 (5.5)	0.67	6.5 (7.1)	2 (7)	0.29
ΔCystatinC(mg/L) [median. (SIQR)]	0.02 (0.09)	0.04 (0.07)	0.24	0.04 (0.1)	0.04 (0.08)	0.55	0.02 (0.09)	0.04 (0.07)	0.32
ΔCys-GFR (ml/min/1.73m²) [median. (SIQR)]	-2.5 (12.2)	-5.8 (6.5)	0.2	-2.6 (12.5)	-6.2 (8.5)	0.60	-2.5 (12.1)	-5.25 (5.8)	0.26
Δ hsCRP (mg/L) [median. (SIQR)]	0.25 (1.5)	0.3 (0.7)	0.78	0.25 (2.2)	0.3 (0.9)	0.26	0.25 (1.4)	0.3 (1.0)	0.49
Δ proBNP (pg/ml) [median. (SIQR)]	-5.2 (21.5)	-13.25 (28.8)	0.12	-6.1 (17.1)	-32.1 (46.1)	0.006	-2.9 (31.1)	-1.5 (27.6)	0.61

All Variables are expressed by median (semi-interquartile range).

Δ: difference between the follow-up level and the baseline level

AR group: Atorvastatin reloading group, Cys: cystatin C; hsCRP, C-reactive protein; Cys-GFR, glomerular filtration calculated using cystatin level; NR group: Non-reloading group, SIQR: semi-interquartile range. ProBNP: Pro Brain natriuretic peptide.

## Discussion

Contrast-induced acute kidney injury (CI-AKI) is a common complication of CM intravascular injection; the physiopathology is complex and not well-understood. Our randomized trial aimed to assess the impact of a reloading dose with 80 mg of atorvastatin on CIN incidence in patients who underwent coronary invasive procedures and who were pre-treated with atorvastatin. The incidence of Scr-based CIN was 10% and the incidence of CYs-based CIN was 31.8% in our trial. In previous studies, the incidence of CYs-CIN ranged between 11 and 28% [20, 23], this is probably due to the high prevalence of diabetes in our population (51%) whereas it was about 30–35% in the other populations.

The mechanisms of CIN are not well-understood. The most recognized mechanisms are renal vasoconstriction, medullary hypoxia, direct tubular cell toxicity of CM, inflammatory mechanisms, and oxidative stress. All these factors lead to epithelial and endothelial cell apoptosis and GFR reduction [3, 9, 24–27]. Several researchers have looked into viable prevention and treatment methods.

The beneficial impact of statins in CIN prevention has been largely demonstrated [14]. Statins have been suggested to enhance endothelial function and reduce oxidative stress and inflammation. They may also reduce the reabsorption of contrast agents in renal tubules [1, 10, 12, 13, 28–37]. Previous studies had shown that loading doses with statins in pre-treated patients before PCI could reduce the myocardial damage and intra-hospital cardiac events, possibly via antioxidant and anti-inflammatory effects, as well as endothelin secretion [29, 37, 38]. Early observational trials and meta-analyses on statin failed to show its beneficial effects on CIN prevention. The methodology was heterogeneous with differences in the definition of CIN [39]. Recently, a few RCTs reported that 80 mg atorvastatin was associated with a significantly lower incidence of CIN in patients undergoing coronary angiology or PCI with chronic kidney disease (eGFR <60 mL/min/ 1.73 m2), in which CIN was defined as CyC increase >10% [20]. Another study enrolling diabetic patients undergoing elective PCI with mild-to-moderate chronic kidney disease (30 < eGFR <90 mL/min/1.73 m2) also revealed the benefit of high-dose (atorvastatin 80 mg) in preventing CIN in such patients [40]. Similarly, in a recent meta-analysis including 6385 patients from 21 RCTs with CKD and naïve of statin, statin loading dose before contrast administration was associated with a significant reduction of CI-AKI risk in CKD patients undergoing cardiac catheterization (odds ratio = 0.46; p <0.05) [13]. All these studies included statin-naïve patients and have already impacted the following guidelines on myocardial revascularization [15]. Thus, we suggested that a reloading dose could also reduce the risk of CIN–and that is why we conducted this study. To our knowledge, this is the first randomized trial that investigates the impact of a reloading dose of statin on CIN incidence in patients on chronic statin therapy. Moreover, most of the studies used changes in creatinine clearance to diagnose CIN incidence. It has been shown that the level of SCr is a result of both GFR and renal tubular secretion, suggesting that changes in SCr underestimate the drop in GFR. Besides, when GFR decreases drastically during the rapid deterioration of renal function, SCr secretion also decreases, and the remaining SCr is distributed in the water of the whole body which leads to a slow rise in SCr (at least 24–48 hours) for the fall in GFR [16, 19]. Cystatin C is produced in all nucleated cells at a constant rate and is freely filtered by the glomerulus and almost completely reabsorbed and catabolized by the proximal tubular cells due to its low molecular weight. Thus, the plasma concentration of Cystatin is determined by glomerular filtration alone without the influence of tubular secretion or its short half-life and only extracellular distribution of Cystatin contributes to the rapid rise and the early attainment, which indicates that this biomarker reflects changes in renal function more quickly and accurately compared with creatinine [17, 18].

Our trial suggested that reloading with high-dose atorvastatin reduces the risk of CIN in diabetic type 2 patients. This beneficial effect could be explained by a reduction in inflammatory reactions after contrast injection, as it was assessed by the significant increase of the hs-CRP level in the NR group and not in the AR group.

Prophylactic treatment with high-dose atorvastatin reduced the incidence of both SCr- and CyC-based CIN in patients with ACS following PCI, which might be attributed to its properties of reducing oxidative stress and inhibiting inflammatory responses.

Reloading with atorvastatin was not superior to maintaining the habitual dose in non-diabetic patients and the overall population. This finding could be explained by the population included in the study, where we already used many means of CIN prevention (chronic statin therapy, rehydration, withdrawal of nephrotoxic drugs, low osmolar contrast products), and it could be difficult to show a benefit from an additional strategy. However, the control group included patients who were already on chronic statins. We recall that in the previous statin studies, the control group included statin-naïve patients.

A recent meta-analysis, including seven RCTs of 4256 participants proved that the risk of developing CIN in patients with CKD pre-treated with statins was significantly lower than that in patients pre-treated with placebo (RR = 0.57. 95%CI = 0.43–0.76. p<0.001); in the subgroup analysis, statin pre-treatment could decrease the risk of CIN in CKD patients with diabetes (RR = 0.54. 95% CI = 0.39–0.76. p<0.001), but not in CKD patients without DM (RR = 0.84. 95% CI = 0.44–1.60. p = 0.606) [8]. These findings were similar to the findings of our trial. Oxidative stress is independently associated with the pathogenesis of diabetic nephropathy. Prolonged hyperglycemia, accumulation of advanced glycation end products, and increased levels of activated transforming growth factor (TGF)-b1 in the glomerular and tubular epithelial cells can result in increased production of ROS, which contributes to oxidative stress. In a recent experimental study, atorvastatin was shown to markedly increase the serum concentration of H_2_S and renal expression of CSE and CBS which contribute to the reduction of oxidative stress [41]. Shehata et al also found in an RCT including only diabetic patients undergoing PCI that atorvastatin 80mg per day for 48 h is associated with decreased incidence of CIN [40].

Paradoxically, Naikuan et al who compared the efficacy of high-dose atorvastatin (40 mg) to conventional-dose atorvastatin (10 mg) on the prevention of CIN in patients with acute coronary syndrome undergoing percutaneous intervention didn’t find a beneficial impact in diabetic groups and they attributed this finding to the low proportion of high-risk patients or lower dose of atorvastatin compared to the studies mentioned above [23].

### Study limitations

The present study had some limitations. Firstly, it was a single-center, single-blinded study. Only the patients were aware of the inclusion group because we didn’t use a placebo for patients in the NR group. Nevertheless, the two doctors who performed the coronary intervention and ensured the follow-up were blinded to the patient’s group. This limitation seems not to have impacted the results since the data of the study did not depend on the psychological state of the patient. Secondly, given that the patients were followed for only 72 hours after the procedure to diagnose CIN, we didn’t evaluate the morbidity associated with CIN.

## Conclusions

Our study was the first randomized trial in the literature that assessed the beneficial effect of 80 mg atorvastatin reloading on CI-AKI in patients pre-treated with this drug at a lower dose. Our population was at high risk with a high prevalence of cardiovascular factors. We did not find a beneficial effect on the overall population. However, the subgroup analysis showed that this intervention reduces the risk of Cys-based CIN in patients with diabetes type 2. Thus, a short-term reloading regimen of atorvastatin could be advised before coronary catheterization in diabetic patients.

## Supporting information

S1 File CONSORT checklist. (DOC)

S2 File Ethic agreement. (PDF)

S3 File Study design. (DOCX)

S4 File Pan-african-trial registry- registration. (PDF)

S5 File Protocol of the study. (DOCX)

S1 Checklist COREQ (COnsolidated criteria for REporting Qualitative research) checklist. (PDF)

S1 Data (XLSX)
