# Supplementary material for: Impact of atorvastatin reload on the prevention of contrast-induced nephropathy in patients on chronic statin therapy: A prospective randomized trial
Source: PLoS One. 2023 May 8;18(5):e0270000. doi: 10.1371/journal.pone.0270000 (PMC10166561; doi:10.1371/journal.pone.0270000)
Supplement: S2 File — (PDF) [file pone.0270000.s002.pdf]

MINISTERE DE LA SANTE
COMITE DE PROTECTION DES PERSONNES SUD
« C.P.P.SUD »

Le Comité de Protection des Personnes Sud « C.P.P.SUD ».a été saisi, le Samedi 08 Février 2020 pour un avis d'éthique, concernant une étude intitulée :

Intérêt des statines dans la prévention de la néphropathie au produit de contraste .

Référence : « CPP SUD N° 0222/2020 »

Cette étude élaboré par : **Dr Mouna Turki.**

- Laboratoire Biochimie du CHU Habib Bourguiba Sfax

Cette étude à été présenté par : **Dr Mouna Turki.**

Après discussion, les membres du Comité ont émis un : **avis favorable**

à la réalisation de ce travail qui obéit aux règles d'éthique de notre pays.

Date
…08/02/2020…

signature du Président

Comité de Protection des Personnes
………C.P.P. SUD…………
Le Président
Professeur Zouhir Bahloul
