# Supplementary material for: Impact of atorvastatin reload on the prevention of contrast-induced nephropathy in patients on chronic statin therapy: A prospective randomized trial
Source: PLoS One. 2023 May 8;18(5):e0270000. doi: 10.1371/journal.pone.0270000 (PMC10166561; doi:10.1371/journal.pone.0270000)
Supplement: S3 File — (DOCX) [file pone.0270000.s003.docx]

**Impact of Atorvastatin Reload on the Prevention of Contrast-Induced Nephropathy**

**in patients on Chronic Statin Therapy: A prospective randomized trial**

**study design**

## Study Population

This is a prospective, randomized, single-blind, controlled trial, implemented in all consecutive patients (older than 18 years), undergoing coronary angiography or percutaneous coronary intervention in our department between June 2020 and September 2020 and who had already been receiving atorvastatin for at least one week, before admission.

We didn’t include in the present study patients with the following criteria: patients admitted because of an acute coronary syndrome in whom a loading dose is recommended according to guidelines, statin-naïve patients, patients who received a statin other than atorvastatin before the procedure, patients already receiving 80 mg atorvastatin, patients requiring dialysis and those with eGFR less than 15 ml/min/ 1.73 m2, patients who were exposed to a contrast medium within 7 days, patients with an allergy to contrast media, patients with cardiogenic shock or severe cardiac insufficiency (left ventricular ejection fraction LVEF <20%), patients with severe liver damage, malignant tumor, infectious disease, or fever, and those who refused to consent. We also excluded the patients who didn't return to get control laboratory tests.

The regional ethics committee approved the study and all participants signed the written informed consent.

## Study Protocol

We randomly assigned all patients to either the Atorvastatin reloading group (AR group) or the Non-reloading group (NR group) according to a computer-generated random series of numbers. Patients in the AR group received oral atorvastatin 80 mg daily one day before and then 3 days after contrast media administration, followed by their habitual dose; patients assigned to the NR group received their habitual dose (atorvastatin 40 mg, 20 mg, 10 mg) without an additional reloading dose.

In accordance with the ESC guidelines, we suspended the nephrotoxic drugs one day before the procedure (aldosterone antagonists, inflammatory inhibitors) in all patients. The renin-angiotensin inhibitors and metformin were withheld if patients showed a moderate CKD (defined by an eGFR <60 mL/min/1.73 m²).

All patients were treated with intravenous hydration with isotonic saline (0.9% sodium chloride) for 12 hours before and 12 hours after the procedure at the rate of 1 ml/Kg/H (0.5 ml/kg/H if LVEF <40% or if the patient suffered from dyspnea) and received the same nonionic dimeric iso-osmolar contrast **media** (Iopromide. ULTRAVIST 300 (300 mg d'Iode/mL). The nurses performed drug delivery and hydration. This designed study is single-blind, therefore, the physicians performing PCI were not aware of the randomization process.

We collected demographics, clinical, and biological data for all patients: age, gender, body mass index (BMI), cardiovascular risk factors, co-morbidities, clinical presentation, the kind of procedure (coronary angiography or PCI), left ventricle systolic function, diastolic dysfunction, and current medication. The details of the procedure were also documented.

## Laboratory Parameters

Blood samples were collected to measure the baseline values of Serum Creatinin (SCr), Cystatin C (Cys), inflammatory factors (high sensitive C-reactive protein [hsCRP]), and pro-BNP on admission, and of course, before the loading dose administration. The post-procedural levels of Cys were measured at 24 hours after the coronary procedure, the SCr, and the hsCRP at 72 hours. Previous studies had shown that the peak of cystatin C elevation occurred by the 24^th^ hour, unlike SCr the peak occurred between the 48^th^ and 72^th^ H (18). Serum levels of creatinine were measured enzymatically and GFR was estimated by the MDRD formula (male: eGFR- Scr=186×(serum creatinine)−1.154×(age)−0.203; female: GFR=186×(serum creatinine)−1.154×(age)−0.203×0.742). Serum cystatin C was measured by nephelometry and eGFR-Cys was estimated by GFR=[74.835/(serum cystatin C (mg/l))] ^1.333^ (19).

## Study End-Points and Definitions

The primary endpoints were the incidence of Cys-based CIN defined as an increase in serum CyC concentration by 10% above the baseline value 24 hours after contrast media administration (17) and the incidence of SCr-based CIN defined as the increase in SCr concentration of 44.2 mmol/L or 25% above baseline within 72 hours after exposure to contrast media.

The secondary end-point was to detect any acute kidney injury by a significant rise in cystatin C level between baseline and 24 hours in the two groups ( AR and NR groups).

We assessed the risk of CIN before the procedure using the Mehran score (20). The risk of CIN was considered low if Mehran’s score =0-5, moderate if Mehran’s score=6-10, High if Mehran’s score>10.

## Statistical Analysis

Statistical analyses were carried out using SPSS software version 23 (SPSS Inc., Chicago. Illinois, the USA). We expressed continuous variables as mean values (±standard deviation [SD]) or medians with interquartile ranges (IQR) and categorical variables as percentages. Normally distributed continuous variables were compared using the Student t-test (independent sample t-test for comparison between the 2 groups, paired sample t-test for self-comparison); non-normally distributed continuous variables were analyzed by non-parametric test (Wilcoxon-Mann-Whitney U test). Categorical data were investigated by the Chi2 or the Fisher exact test.

Given the lack of similar studies in the literature, we carried out a pre-survey on 20 patients to determine the number of subjects needed. It was speculated that the incidence of CyC-based CIN was 36% in the NR group. We hypothesized the additional loading dose of Atorvastatin in the AR group could reduce the incidence of CyC-based CIN to 15%. Thus, the calculated sample size was at least 51 individuals for each group to get 80% power with a significance level of 0.05 (for the unilateral test). We have included an additional 10% of the workforce calculated taking into account the risk of loss of follow-up.
