# Supplementary material for: Impact of atorvastatin reload on the prevention of contrast-induced nephropathy in patients on chronic statin therapy: A prospective randomized trial
Source: PLoS One. 2023 May 8;18(5):e0270000. doi: 10.1371/journal.pone.0270000 (PMC10166561; doi:10.1371/journal.pone.0270000)
Supplement: S4 File — (PDF) [file pone.0270000.s004.pdf]

Pan African Clinical Trials Registry

South African Medical Research Council, South African Cochrane Centre
PO Box 19070, Tygerberg, 7505, South Africa
Telephone: +27 21 938 0506 / +27 21 938 0834 Fax: +27 21 938 0836
Email: pactradmin@mrc.ac.za Website: www.pactr.org

Trial no.:		Date of Approval:	
Trial Status:			

TRIAL DESCRIPTION

Public title	Impact of ATOrvastatin REload on the Prevention of Contrast-Induced Nephropathy in patients on Chronic Statin Therapy: A prospective randomized trial (ATORE-CIN)
Official scientific title	Impact of Atorvastatin Reload on the Prevention of Contrast-Induced Nephropathy
Brief summary describing the background and objectives of the trial	high dose of atorvastatine before coronary procedures reduces the risk of Contrast-Induced Nephropathy in statine naive patients. This trial aimed to assess the efficacy of Atorvastatin reload on the prevention of Contrast-Induced Nephropathy in patients pre-treated with this statin and undergoing coronary catheterization.
Type of trial	RCT
Acronym (If the trial has an acronym then please provide)	ATORECIN
Disease(s) or condition(s) being studied	renal diseases
Sub-Disease(s) or condition(s) being studied	
Purpose of the trial	Prevention
Anticipated trial start date	01/06/2020
Actual trial start date	30/09/2020
Anticipated date of last follow up	15/10/2020
Actual Last follow-up date	15/10/2020
Anticipated target sample size (number of participants)	102
Actual target sample size (number of participants)	110
Recruitment status	Completed
Publication URL	

Secondary Ids	Issuing authority/Trial register

STUDY DESIGN

Intervention assignment	Allocation to intervention	If randomised, describe how the allocation sequence was generated	Describe how the allocation sequence/code was concealed from the person allocating the participants to the intervention arms	Masking	If masking / blinding was used
Factorial: participants randomly allocated to either no, one, some or all interventions simultaneously	Randomised	Simple randomization using a randomization table created by a computer software program	Allocation was determined by the holder of the sequence who is situated off site	Open-label(Masking Not Used)	

INTERVENTIONS

Intervention type	Intervention name	Dose	Duration	Intervention description	Group size	Nature of control
Experimental Group	Reload patients on chronic statin therapy	80 mg of atorvastatin	one day before contrast injection and three days after	Reload patients on chronic statin therapy with high dose of atorvastatin	55	

	with high dose of atorvastatin					
Control Group	no relaod statin	nothing	nothing	contrast injection without statin reloading	55	Active-Treatment of Control Group

ELIGIBILITY CRITERIA

List inclusion criteria	List exclusion criteria	Age Category	Minimum age	Maximum age	Gender
This is a prospective, randomized, single-blind, controlled trial, implemented in all consecutive patients (older than 18 years), undergoing coronary angiography or percutaneous coronary intervention in our department between June 2020 and September 2020 and who had already been receiving atorvastatin for at least one week, before admission.	*patients admitted because of an acute coronary syndrome in whom a loading dose is recommended according to guidelines, *statin-naïve patients, *patients who received a statin other than atorvastatin before the procedure, *patients already receiving 80 mg atorvastatin, patients requiring dialysis and those with eGFR less than 15 ml/min/ 1.73 m2, *patients who were exposed to a contrast medium within 7 days, patients with an allergy to contrast media, *patients with cardiogenic shock or severe cardiac insufficiency (left ventricular ejection fraction LVEF <20%), *patients with severe liver damage, malignant tumor, infectious disease, or fever, *and those who refused to consent. * patients who didn't return to get control laboratory tests	Adult: 19 Year-44 Year	18 Year(s)	100 Year(s)	Both

ETHICS APPROVAL

Has the study received appropriate ethics committee approval	Date the study will be submitted for approval	Date of approval	Name of the ethics committee
Yes		01/04/2020	CPP sud

Ethics Committee Address

Street address	City	Postal code	Country
hedi chaker hospital	sfax	3029	Tunisia

OUTCOMES

Type of outcome	Outcome	Timepoint(s) at which outcome measured
Primary Outcome	the incidence of Cys-based CIN defined as an increase in serum CyC concentration by 10% above the baseline value 24 hours after contrast media administration (17) and the incidence of SCr-based CIN defined as the increase in SCr the concentration of 44.2 mmol/L or 25% above baseline within 72 hours after exposure to contrast media.	24 hours and 72 hours
Secondary Outcome	The secondary end-point was to detect any acute kidney injury by a significant rise in cystatin C level between baseline and 24 hours in the two groups (AR and NR groups). We assessed the risk of CIN before the procedure using the Mehran score (20). The risk of CIN was considered low if Mehran’s score =0-5, moderate if Mehran’s score=6-10, High if Mehran’s score>10.	24 h and 72 h

RECRUITMENT CENTRES

Name of recruitment centre	Street address	City	Postal code	Country
cardiology departement of sfax	service de cardiologie	sfax	3029	Tunisia

FUNDING SOURCES

Name of source	Street address	City	Postal code	Country
hedi chaker hospital	hedi chaker hospital	sfax	3029	Tunisia

SPONSORS

Sponsor level	Name	Street address	City	Postal code	Country	Nature of sponsor
Primary Sponsor	hedi chaker hospital	hedi chaker hospital	sfax	3029	Tunisia	Hospital

COLLABORATORS

Name	Street address	City	Postal code	Country
Omar masmoudi	hedi chaker hospital	sfax	3029	Tunisia

CONTACT PEOPLE

Role	Name	Email	Phone	Street address
**Scientific Enquiries**	Rania Hammami	raniahammami@yahoo.fr	0021624056985	heddi chaker hospital
**City**	**Postal code**	**Country**	**Position/Affiliation**	
sfax	3029	Tunisia	professor	
**Role**	**Name**	**Email**	**Phone**	**Street address**
**Principal Investigator**	omar masmoudi	masmoudiomar@hotmail.com	0021626823843	heddi chaker hospital
**City**	**Postal code**	**Country**	**Position/Affiliation**	
sfax	3029	Tunisia	fellow	
**Role**	**Name**	**Email**	**Phone**	**Street address**
**Public Enquiries**	rania hammami	raniahammami@yahoo.fr	0021624056985	hedi chaker hospital
**City**	**Postal code**	**Country**	**Position/Affiliation**	
sfax	3029	Tunisia	professor	

REPORTING

Share IPD	Description	Additional Document Types	Sharing Time Frame	Key Access Criteria
Yes	Background: This trial aimed to assess the efficacy of Atorvastatin reload on the prevention of CIN in patients pre-treated with this statin and undergoing coronary catheterization. Methods: This is a prospective randomized controlled study including patients on chronic atorvastatin therapy. We compared two groups: The Atorvastatin Reloading group (AR group) including patients who received 80 mg of atorvastatin one day before and 3 days after the coronary procedure and the Non-reloading group (NR group). The primary endpoints were the incidence of cystatin (Cys)-based CIN and Creatinine (Scr)-based CIN. Results: We randomly included 56 patients in the AR group and 54 patients in the NR group. The baseline characteristics of the 2 groups were similar. Serum creatinine (SCr)-based CIN occurred in 11.1% in the NR group and 8.9% in the AR group without any significant difference. Cys-based CIN occurred in 37% in the NR group and 26.8% in the AR group without any significant difference. The subgroups analysis showed that high dose reloading had significantly reduced the CYC-based CIN risk in patients with type 2 diabetes (43.5% vs 18.8%, RR = 0.43. CI 95% [0.18-0.99])). Cystatin C had significantly increased between baseline and at 24 hours in the NR group (0.96 vs 1.05, p=0.001) but not in the AR group (0.94 vs 1.03,p=0.206). Conclusions: Our study demonstrated that prophylactic reloading with high-dose atorvastatin reduced the risk of CyC-based CIN in diabetic type 2 patients and reduced the risk of AKI induced by contrast injection.	Study Protocol	no	no

URL	Results Available	Results Summary	Result Posting Date	First Journal Publication Date
	No			
**Result Upload 1:**	**Result Upload 2:**	**Result Upload 3:**	**Result Upload 4:**	**Result Upload 5:**

Result URL Hyperlinks	Link To Protocol
Result URL Hyperlinks	

Changes to trial information
