# Supplementary material for: Impact of atorvastatin reload on the prevention of contrast-induced nephropathy in patients on chronic statin therapy: A prospective randomized trial
Source: PLoS One. 2023 May 8;18(5):e0270000. doi: 10.1371/journal.pone.0270000 (PMC10166561; doi:10.1371/journal.pone.0270000)
Supplement: S5 File — (DOCX) [file pone.0270000.s005.docx]

# Protocol of the study

# Background

Contrast-induced nephropathy (CIN) is a common complication occurring in 5 to 11% of angiographic procedures [1–4]. The CIN incidence has varied widely across studies as its definition is not the same throughout the literature [5]. CIN is the third leading cause of hospital-acquired acute kidney injury (AKI), accounting for 12% of all cases, next to hypovolemia (42%) and postoperative renal injury (18%) [6]; it accounts for up to 30% of acute kidney injury in hospitalized patients [7]. This complication is strongly associated with significantly increased mortality, extended hospitalization periods, and additional costs [8]. Given the increase in the number of coronary intervention procedures in the last decades and the difficulties to determine CIN mechanisms, many protective measures have been assessed in randomized and observational studies. The most known predictors of CIN occurrence according to the literature are advanced age, diabetes, use of angiotensin-converting enzyme inhibitor/angiotensin receptor blocker before the procedure, high baseline creatinine, the contrast type, hypertension, and left ventricular systolic dysfunction [1, 3, 7, 9]. Paradoxically, Statin pretreatment has been identified as a protector against CIN after PCI in observational studies [10, 11]. Recently, Randomized Controlled Trials (RCTs) including statin naïve patients showed that a loading dose of statin before catheterization reduces the risk of CIN significantly [12, 13]. In a recent large meta-analysis of 124 trials and 28240 patients, comparing 10 strategies of CIN prevention: saline, statin, and other strategies like N-acetylcysteine (NAC), sodium bicarbonate (NaHCO_3_), NAC+NaHCO_3_, ascorbic acid, xanthine, dopaminergic agent, peripheral ischemic preconditioning, and natriuretic peptide; compared with saline, the risk of CIN was significantly reduced by using statin (odds ratio [OR]= 0.42; 95% confidence interval [CI], 0.26-0.67). The benefit of statin therapy was consistent across multiple sensitivity analyses, whereas the efficacy of all the other strategies was questioned by restricting the analysis to high-quality trials [14]. Thus, the ESC guidelines on myocardial revascularization have already supported pre-treatment with high dose statins in statin-naïve patients since 2018, to prevent CIN ( Class IIa, evidence level A) [15]. Up to date, there is still no evidence about the impact of statin reloading in patients on chronic statin therapy. Moreover, in most trials, we used a glomerular filtration rate (GFR) assessed by creatinine blood level. However, unlike cystatin C, creatinine is significantly affected by muscle mass (hence, sex or age), race, or diet. Furthermore, many studies proved that cystatin C, a small protein produced throughout the body by all nucleus cells, could be a more reliable and sensitive marker of kidney function and could be potentially used to generate a more precise estimate of GFR than creatinine [16–18]. Thus, we aimed in this trial to assess the impact of an additional loading dose of atorvastatin in patients who had already been on this drug on CIN incidence using measurement of Cystatin C level in blood.

# Methods

## Study Population

*Inclusion criteria*

This was an interventional prospective, randomized, single-blind, controlled trial, implemented in all consecutive patients (older than 18 years), undergoing coronary angiography or percutaneous coronary intervention in our department between June 2020 and September 2020 and who had already been receiving atorvastatin for at least one week, before admission.

*Non-inclusion criteria*

We didn’t include in the present study patients with the following criteria: patients admitted because of an acute coronary syndrome in whom a loading dose is mandatory according to guidelines, statin-naïve patients, patients who received a statin other than atorvastatin before the procedure, patients already receiving 80 mg atorvastatin, patients requiring dialysis and those with eGFR less than 15 ml/min/ 1.73 m2, patients who were exposed to a Contrast Medium (CM) within 7 days, patients with an allergy to contrast media, patients with cardiogenic shock or severe cardiac insufficiency (left ventricular ejection fraction LVEF <20%), patients with severe liver damage, malignant tumor, infectious disease, or fever, and those who refused to consent. We also excluded the patients who didn't return to get control laboratory tests.

The regional ethics committee (The Committee of Protection of the Persons in the South of the country: CPP South) approved the study and all participants signed the written informed consent.

The protocol of the study was registered on the Pan-African Clinical Registry (PACTR). The registration number of the study is: PACTR202110707328144.

## Study Protocol

We randomly assigned all patients to either the Atorvastatin Reloading group (AR group) or the Non-Reloading group (NR group) according to a computer-generated random series of numbers. The randomization occurs one day before the coronary procedure. Patients in the AR group received oral atorvastatin 80 mg daily one day before and then 3 days after contrast media administration, followed by their habitual dose; patients assigned to the NR group received their habitual dose (atorvastatin 40 mg, 20 mg, 10 mg) without an additional reloading dose.

In accordance with the ESC guidelines, we suspended the nephrotoxic drugs one day before the procedure (aldosterone antagonists, inflammatory inhibitors) in all patients. The renin-angiotensin inhibitors and metformin were withheld if patients showed a moderate CKD (defined by an eGFR <60 mL/min/1.73 m²) [15].

All patients were treated with intravenous hydration with isotonic saline (0.9% sodium chloride) for 12 hours before and 12 hours after the procedure at the rate of 1 ml/Kg/H (0.5 ml/kg/H if LVEF <40% or if the patient suffered from dyspnea) and received the same nonionic dimeric iso-osmolar Contrast Media (CM) (Iopromide. ULTRAVIST 300 (300 mg d'Iode/mL). The nurses performed drug delivery and hydration. This designed study was single-blind. The physician who performed the coronary procedure was blinded to the patient’s group, but the patient was aware of his group.

The nurses ( 3 females) collected demographics, clinical, and biological data for all patients: age, gender, body mass index (BMI), cardiovascular risk factors, co-morbidities, clinical presentation, the kind of procedure (coronary angiography or PCI), left ventricle systolic function, and current medication. No relationship was established prior to the study commencement. The interview was not repetad. The details of the procedure were also documented. All the data were collected in a CRF that was not returned to the participant. The participants didn’t provide feedback on the findings. Data saturation was not discussed.

## Laboratory Parameters

Blood samples were collected to measure the baseline values of Serum Creatinin (SCr), Cystatin C (Cys), inflammatory factors (high sensitive C-reactive protein [hsCRP]), and pro-BNP on admission, and of course, before the loading dose administration. The post-procedural levels of Cys were measured 24 hours after the coronary procedure, the SCr, and the hsCRP at 72 hours. Previous studies had shown that the peak of cystatin C elevation occurred by the 24^th^ hour, unlike SCr the peak occurred between the 48^th^ and 72^nd^ H [19]. Serum levels of creatinine were measured enzymatically and GFR was estimated by the MDRD formula (male: eGFR- Scr=186×(serum creatinine)−1.154×(age)−0.203; female: GFR=186×(serum creatinine)−1.154×(age)−0.203×0.742). Serum cystatin C was measured by nephelometry and eGFR-Cys was estimated by GFR=[74.835/(serum cystatin C (mg/l))] ^1.333^ [20].

## Study End-Points and Definitions

The primary endpoints were the incidence of Cys-based CIN defined as an increase in serum CyC concentration by 10% above the baseline value 24 hours after contrast media administration [18] or the incidence of SCr-based CIN defined as the increase in SCr concentration of 44.2 mmol/L or 25% above baseline within 72 hours after exposure to contrast media according to the KDIGO definition [21].

The secondary end-point was to detect any acute kidney injury by a significant rise in cystatin C level between baseline and 24 hours in the two groups (Δ cystatin).

The changes in renal biomarkers (Δ biomarkers) are defined as the difference between the follow-up level and the baseline level. These changes were compared between the two groups

(AR and NR groups).

We assessed the risk of CIN before the procedure using the Mehran score [22]. It was first described by Roxana Mehran et al, in 2004, in a randomized study including 8351 patients undergoing PCI and aiming to determine predictors of CIN after PCI [22]. This score includes eight parameters (hypotension, [intra-aortic balloon pump](https://www.sciencedirect.com/topics/medicine-and-dentistry/intra-aortic-balloon-pump), [congestive heart failure](https://www.sciencedirect.com/topics/medicine-and-dentistry/congestive-heart-failure), [chronic kidney disease](https://www.sciencedirect.com/topics/medicine-and-dentistry/chronic-kidney-disease), diabetes, age >75 years, anemia, and volume of contrast) and predicts the incidence of CIN with good discriminative power (*c*statistic = 0.67) [22]. The risk of CIN was considered low if Mehran’s score = 0 to 5, moderate if Mehran’s score = 6 to 10, and high if Mehran’s score>10.

## Statistical Analysis

Statistical analyses were carried out using SPSS software version 23 (SPSS Inc., Chicago. Illinois, the USA). We expressed categorical variables as percentages, and continuous variables as mean values (±standard deviation [SD]) when the distribution was normal or medians with semi-interquartile ranges (SIQR) when it was not normal. Normally distributed continuous variables were compared using the Student t-test (independent sample t-test for comparison between the 2 groups, paired sample t-test for self-comparison); non-normally distributed continuous variables were analyzed by non-parametric test (Mann-Whitney U test for independent series and Wilcoxon test for paired series ). When the application conditions were validated, categorical data were analyzed using the Chi2 test of Pearson, otherwise Fisher exact test.

Given the lack of similar studies in the literature, we carried out a pre-survey on 20 patients to determine the number of subjects needed. It was speculated that the incidence of CyC-based CIN was 36% in the NR group. We hypothesized the additional loading dose of Atorvastatin in the AR group could reduce the incidence of CyC-based CIN to 15%. Thus, the calculated sample size was at least 51 individuals for each group to get 80% power with a significance level of 0.05 (for the unilateral test). We have included an additional 10% of the workforce calculated taking into account the risk of loss of follow-up.

The number needed to treat to prevent one event was calculated according to this formula: NNT= 1/ARR with ARR = Absolute Risk Reduction.

References

Katsiki N, Fonseca V, Mikhailidis DP. Contrast-induced acute kidney injury in diabetes mellitus: Clinical relevance and predisposing factors. Could statins be of benefit? *J Diabetes Complications* 2018; 32: 982–984.

[2] Chandiramani R, Cao D, Nicolas J, et al. Contrast-induced acute kidney injury. *Cardiovasc Interv Ther* 2020; 35: 209–217.

[3] Leisman S. Radiocontrast Toxicity. *Adv Chronic Kidney Dis* 2020; 27: 50–55.

[4] Novak JE, Handa R. Contrast Nephropathy Associated with Percutaneous Coronary Angiography and Intervention. *Cardiol Clin* 2019; 37: 287–296.

[5] Budano C, Levis M, D’Amico M, et al. Impact of contrast-induced acute kidney injury definition on clinical outcomes. *Am Heart J* 2011; 161: 963–971.

[6] Nash K, Hafeez A, Hou S. Hospital-acquired renal insufficiency. *Am J Kidney Dis* 2002; 39: 930–936.

[7] Fähling M, Seeliger E, Patzak A, et al. Understanding and preventing contrast-induced acute kidney injury. *Nature Reviews Nephrology* 2017; 13: 169–180.

[8] Zhou YL, Chen LQ, Du XG. Efficacy of short-term moderate or high-dose statin therapy for the prevention of contrast-induced nephropathy in high-risk patients with chronic kidney disease: Systematic review and meta-analysis. *Clinics*; 76. Epub ahead of print 2021. DOI: 10.6061/CLINICS/2021/E1876.

[9] Vlachopanos G, Schizas D, Hasemaki N, et al. Pathophysiology of Contrast-Induced Acute Kidney Injury (CIAKI). *Curr Pharm Des* 2019; 25: 4642–4647.

[10] Lin M, Xu T, Zhang W, et al. Effect of statins on post-contrast acute kidney injury: a multicenter retrospective observational study. *Lipids Health Dis*; 20. Epub ahead of print 1 December 2021. DOI: 10.1186/S12944-021-01489-7.

[11] Moitinho MS, Santos ES, Caixeta AM, et al. Contrast-Induced Nephropathy in patients submitted to percutaneous coronary intervention: an integrative review. 2020; 73: e20200190.

[12] Cheungpasitporn W, Thongprayoon C, Kittanamongkolchai W, et al. Periprocedural effects of statins on the incidence of contrast-induced acute kidney injury: A systematic review and meta-analysis of randomized controlled trials. *Ren Fail* 2015; 37: 664–671.

[13] Zhou X, Dai J, Xu X, et al. Comparative Efficacy of Statins for Prevention of Contrast-Induced Acute Kidney Injury in Patients With Chronic Kidney Disease: A Network Meta-Analysis. *Angiology* 2019; 70: 305–316.

[14] Giacoppo D, Gargiulo G, Buccheri S, et al. Preventive Strategies for Contrast-Induced Acute Kidney Injury in Patients Undergoing Percutaneous Coronary Procedures: Evidence From a Hierarchical Bayesian Network Meta-Analysis of 124 Trials and 28 240 Patients. *Circ Cardiovasc Interv*; 10. Epub ahead of print 1 May 2017. DOI: 10.1161/CIRCINTERVENTIONS.116.004383.

[15] Neumann FJ, Sousa-Uva M, Ahlsson A, et al. 2018 ESC/EACTS Guidelines on myocardial revascularization. *Eur Heart J* 2019; 40: 87–165.

[16] Banda J, Duarte R, Dix-Peek T, et al. Biomarkers for diagnosis and prediction of outcomes in contrast-induced nephropathy. *Int J Nephrol* 2020; 2020: 1–11.

[17] Gu G, Yu N, Zhou Y, et al. Elevation of preoperative cystatin C as an early predictor of contrast-induced nephropathy in patients receiving percutaneous coronary intervention. *Singapore Med J* 2021; 1–16.

[18] Briguori C, Visconti G, Rivera N V., et al. Cystatin C and contrast-induced acute kidney injury. *Circulation* 2010; 121: 2117–2122.

[19] D’Amore C, Nuzzo S, Briguori C. Biomarkers of Contrast-Induced Nephropathy:: Which Ones are Clinically Important? *Interventional Cardiology Clinics* 2020; 9: 335–344.

[20] Quintavalle C, Fiore D, De Micco F, et al. Impact of a high loading dose of atorvastatin on contrast-induced acute kidney injury. *Circulation* 2012; 126: 3008–3016.

[21] Khwaja A. KDIGO clinical practice guidelines for acute kidney injury. *Nephron - Clinical Practice*; 120. Epub ahead of print October 2012. DOI: 10.1159/000339789.

[22] Mehran R, Aymong ED, Nikolsky E, et al. A simple risk score for prediction of contrast-induced nephropathy after percutaneous coronary intervention: Development and initial validation. *J Am Coll Cardiol* 2004; 44: 1393–1399.
